# Adoptive NK Cell Therapy - a Beacon of Hope in Multiple Myeloma Treatment

**DOI:** 10.3389/fonc.2023.1275076

**Published:** 2023-11-03

**Authors:** Son Hai Vu, Ha Hong Pham, Thao Thi Phuong Pham, Thanh Thien Le, Manh-Cuong Vo, Sung-Hoon Jung, Je-Jung Lee, Xuan-Hung Nguyen

**Affiliations:** ^1^ Hi-Tech Center and Vinmec-VinUni Institute of Immunology, Vinmec Healthcare System, Hanoi, Vietnam; ^2^ Research Center for Cancer Immunotherapy, Chonnam National University Hwasun Hospital, Hwasun, Jeollanamdo, Republic of Korea; ^3^ Department of Hematology-Oncology, Chonnam National University Hwasun Hospital and Chonnam National University Medical School, Hwasun, Jeollanamdo, Republic of Korea; ^4^ College of Health Sciences, VinUniversity, Hanoi, Vietnam

**Keywords:** multiple myeloma, chimeric antigen receptor, adoptive NK cell therapy, metabolic engineering, banked cells, clinical trials

## Abstract

Major advances in the treatment of multiple myeloma (MM) have been achieved by effective new agents such as proteasome inhibitors, immunomodulatory drugs, or monoclonal antibodies. Despite significant progress, MM remains still incurable and, recently, cellular immunotherapy has emerged as a promising treatment for relapsed/refractory MM. The emergence of chimeric antigen receptor (CAR) technology has transformed immunotherapy by enhancing the antitumor functions of T cells and natural killer (NK) cells, leading to effective control of hematologic malignancies. Recent advancements in gene delivery to NK cells have paved the way for the clinical application of CAR-NK cell therapy. CAR-NK cell therapy strategies have demonstrated safety, tolerability, and substantial efficacy in treating B cell malignancies in various clinical settings. However, their effectiveness in eliminating MM remains to be established. This review explores multiple approaches to enhance NK cell cytotoxicity, persistence, expansion, and manufacturing processes, and highlights the challenges and opportunities associated with CAR-NK cell therapy against MM. By shedding light on these aspects, this review aims to provide valuable insights into the potential of CAR-NK cell therapy as a promising approach for improving the treatment outcomes of MM patients.

## 1 Introduction

Multiple myeloma (MM) is the second most common hematologic malignancy characterized by the abnormal proliferation of clonal malignant plasma cells in the bone marrow. This results in an overabundance of monoclonal paraprotein (M protein) in the serum and/or urine, causing renal dysfunction, anemia, hypercalcemia, and lytic bone disease, which are the hallmarks of MM (1). The treatment paradigm of MM has significantly evolved with the introduction of novel agents such as proteasome inhibitors, immunomodulatory drugs, and monoclonal antibodies (mAbs). These advancements have led to improved responses and prolonged overall survival (2). However, despite these improvements, most patients with MM eventually experience relapse and succumb to the disease, emphasizing the urgent need for new strategies that offer long-term survival. Given the potential role of natural killer (NK) cells in the pathogenesis and recent treatment successes of MM, NK cell-based immunotherapy merits further exploration.

NK cells are innate lymphocytes capable of rapidly killing malignant cells, including MM, without prior sensitization and gene rearrangement to acquire antigen-specific receptors (3). NK cells employ death ligands and degranulation to eliminate target cells, in which their cytotoxic responses are regulated by the fine-tuned balance between a complex array of inhibitory and activating receptors (4). In MM, NK cell functionality and immunity are negatively regulated by myeloma cells and their immunosuppressive microenvironmental factors (5). The diminished activity and the reduced number of NK cells isolated from advanced-stage MM are associated with adverse prognostic factors (6).

The development of CAR-T cell therapy has demonstrated striking success in treating lymphoid malignancies, including MM (7–10). The breakthrough of CAR technology has given rise to numerous CAR-expressing immune cell-based clinical trials worldwide.

The two current FDA-approved CAR-T cell products for MM, Idecabtagene Vicleucel (Abecma) and ciltacabtagene autoleucel (Carvykti), are autologous cellular therapy, thus limiting its use to patients who have a sufficient number of functional T cells. Given the limited durability of CAR-T therapy responses in multiple myeloma and a high relapse rate of 40% to 60% (11), developing alternative immunotherapeutic treatments is necessary. Allogeneic CAR-T cell products can be produced to overcome cell insufficiency; however, this approach may be associated with severe side effects, such as cytokine release syndrome (CRS), neurotoxicity, and graft-versus-host disease (GvHD). Additionally, the cumbersome CAR-T cell manufacturing process often results in delays during cell collection, production, and delivery, negatively affecting patient outcomes and limiting accessibility.

Building upon the success of the CAR-T cell strategy, CAR-NK cell therapy has emerged as a promising candidate for universal cellular immunotherapy, offering improved safety, efficacy, and tolerability compared to autologous CAR-T cell strategies (11, 12). Notably, NK cells eliminate target cells in an MHC-unrestricted manner, making NK cell-based therapy less likely to cause GvHD, a common complication of allogeneic cell transfers. NK cells possess inherent antitumor responses, including ADCC and degranulation. Thus, incorporating CAR further enhances NK cell cytotoxicity by directing CAR-NK cells toward target cells. Unlike CAR-T cells, CAR-NK cells produce a different set of cytokines (IFN-γ and GM-CSF), attributed to the lower incidence of CRS and neurotoxicity observed in subjects treated with CAR-NK cells (13). Indeed, allogeneic CAR-NK cells have shown antitumor efficacy and a high safety profile in patients with hematological malignancies (13).

In this review, we provide a comprehensive overview of the CAR-NK approach, highlighting updated strategies to enhance NK cell potency and persistence, focusing on MM. We discuss ongoing clinical trials, advancements in NK cell manufacturing, and the potential translation of these developments into therapeutic applications. By capitalizing on the distinctive attributes of CAR-NK cells and leveraging the experience gained from CAR-T cell therapy, we envision a promising future for CAR-NK immunotherapy in MM and beyond.

## 2 Adoptive NK cell therapy

### 2.1 Principle of CAR generation

**Figure 1:**
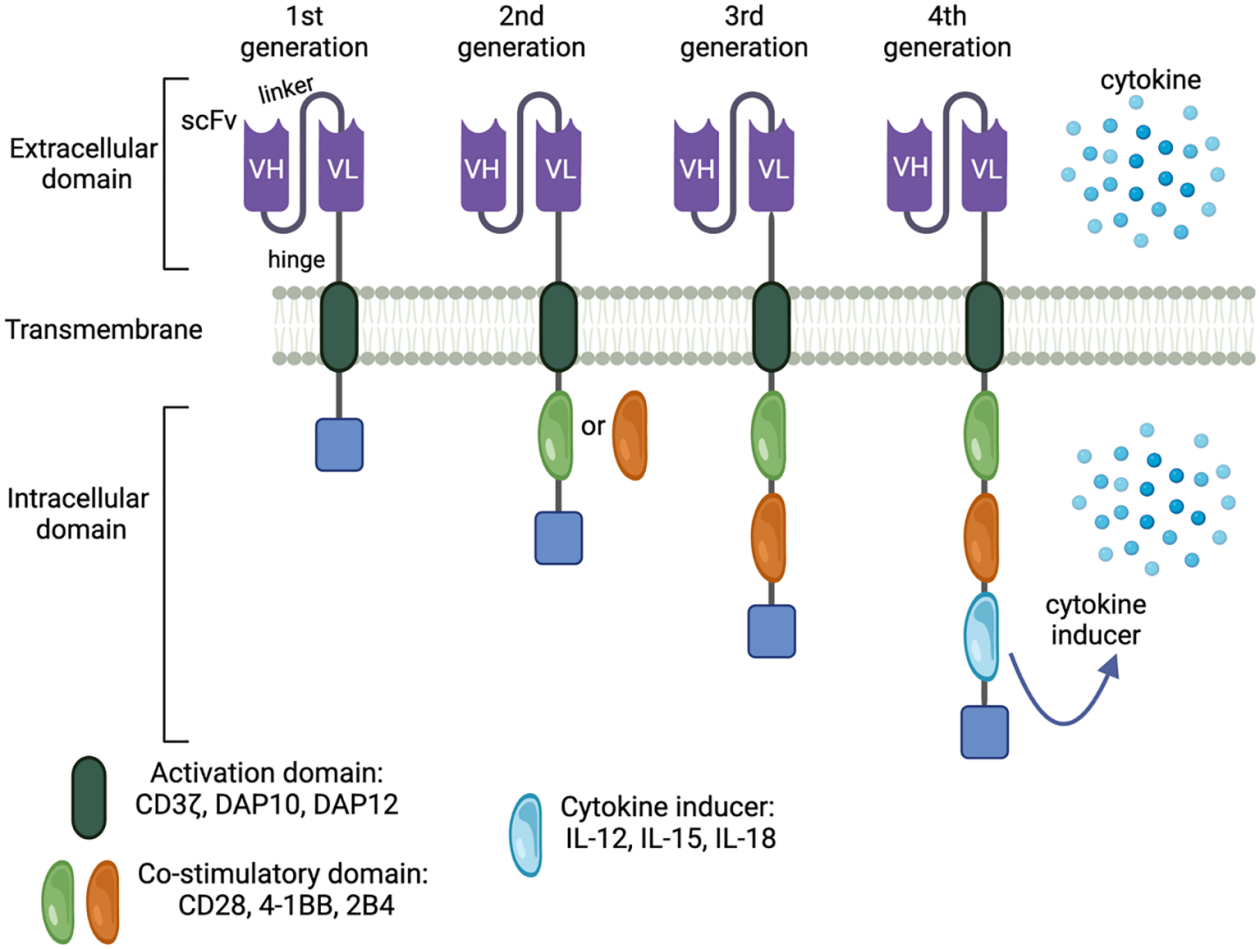
Generation of CAR constructs. Legend 1: CAR technology has been intensively innovated, resulting in increasingly effective and targeted antitumor responses. There are 4 CAR generations characterized by the complexity of the modular system. The first generation is the simplest and also least effective. Adding costimulatory molecules and payloads augments CAR-mediated antitumor immunity by boosting effector cell activation, expansion, and durability, forming the next CAR generations.

Adoptive immune cell transfer has emerged as a groundbreaking approach that harnesses the power of the patient’s immune system and genetic engineering to specifically eliminate tumor cells. The introduction of CARs into immune effector cells has revolutionized the field of cancer immunotherapy, enabling these engineered cells to target specific cancer cells based on antigen recognition. The advent of retroviral vectors in the 1990s paved the way for T-cell engineering, leading to the development of the first generation of CAR-T cells (14). These first-generation CARs exhibit antigen recognition but have limited clinical effectiveness due to the poor activation and proliferation of those CAR-expressing T cells. Early chimeric receptors with only TCR ζ-chain intracellular sequences struggled to fully activate naive T cells, as they lacked necessary costimulatory signals (15, 16). Subsequently, integrating a co-stimulatory moiety into CAR constructs significantly enhanced T-cell functionality, laying the foundation for the successful CAR-based immunotherapy. Second-generation CAR incorporates an extra costimulatory signaling domain, such as CD28 or 4-1BB (CD137), to enhance T cell activation, persistence, and antitumor activity (17, 18) (**Figure 1**). Furthermore, T cells are endowed with more robust antitumor activities when two costimulatory domains are integrated into the cassettes forming third-generation CAR (19). These advancements have also been translated to CAR-NK cells, where incorporating costimulatory domains significantly enhances NK cell proliferation and effector function (20).

Further advancements in CAR design have led to the development of fourth-generation CARs, which involve the integration of interleukins (i.e., IL-12/15/18/21) into 3^rd^ generation CAR. This innovation enables CAR-expressing cells to produce autocrine interleukins, enhancing effector functions and homing to target sites (21) (**Figure 1**).

### 2.2 Engineering CAR to enhance antitumor activity

#### 2.2.1 Advances in engineering CAR components

CAR technology was initially developed for T cells, thus transducing a T cell-specific CAR cassette into other effector cells may not yield desirable antitumor responses due to the distinct functions of each immune cell class. The success of CAR-based cellular therapies against malignant diseases is primarily attributed to the unique design of synthetic constructs. Various design strategies have been employed to address this challenge, such as TanCARs, dual CARs, AND-gate CARs, and iCARs (22). Given the modular structure of CAR, engineering each module may improve the effector functions of transduced cells.

CD8α, CD28 extracellular domains, DAP12, or IgG-based hinges are usually used in current CAR-NK constructs due to their edges, such as stability and flexibility. Balancing extracellular domain-mediated adverse events and signal strength is also very sophisticated since the complete understanding of NK-specific extracellular domain remains elusive (23–25).

CARs with multi-specificity scFv structures have been developed to target different epitopes, overcoming immune selection and escape in tumors with heterogeneous antigen expression (26). The Fv configuration can significantly affect the efficiency and stability of CAR expression on immune cells, as some CARs fail to bind to the antigen due to aggregation at the cell surface. Therefore, stabilizing the Fv structure through the complementarity-determining region (CDR)-grafting renders scFvs with enhancing antigen specificity and affinity (27).

The size of transgenes matters when more than one protein must be expressed in the cellular therapy to elicit effective antitumor responses (28). Another approach to improve CAR target recognition is using nanobodies, which are small-size single-domain antibodies derived from heavy-chain antibodies found in certain animal species such as camels and sharks (29). Nanobodies, typically the variable domain on a heavy chain (VHH) of camelid heavy-chain antibodies, possess clear advantages in size, stable physiochemical properties, and affinity. Furthermore, VHH exhibits high solubility due to the absence of a light chain (30). Importantly, camelid VHH shares significant homology with human-heavy variable fragments, making them superior to conventional antibodies derived from mice or other animals. Replacing scFvs with nanobodies in CAR construction would be a rational strategy to enhance the CAR-mediated tumor-killing capacity of effector cells. To simplify CAR constructs used in T cells targeting MM, single heavy-chain-only domains are generated to substitute for traditional scFvs of B-cell maturation antigen (BCMA), the latter is significantly larger and less immunogenic (28). Interestingly, NK cells transduced with CARs containing anti-BCMA and anti-GPRC5D VHHs also yielded sustained anti-MM responses (31–33).

Nanobodies-based CAR-T approaches targeting antigens of either liquid or solid tumors, such as VEGFR2, HER2, TAG-72, PSMA, GPC2, CD38, CD33, CD7, MUC1, EGFR, CD20, PD-L1, EIIIB, CD105, and BCMA, have demonstrated potential translational outcomes (34). For instance, targeting the immune checkpoint PD-L1 using nanobody-based CAR-T cells significantly reduced tumor growth and prolonged survival. In addition, directing nanobodies against other components of the tumor microenvironment (TME), including extracellular matrix (ECM) and neovasculature, rather than tumor-associated antigens, may be a promising therapeutic approach. Indeed, CAR-T cells incorporating nanobodies specific to anti-EIIIB fibronectin exhibited enhanced tumor infiltration, induction of necrosis, and reduced stromal ECM and neovasculature in mouse models, leading to delayed tumor growth (35). Encouraging clinical outcomes have been observed with VHH-based BCMA-targeted CAR-T cell therapy for MM, with an overall response rate of 88%, and complete response in 68% (39/57) of patients, while 63% (36/57) of refractory/relapsed (R/R) MM patients showed negative minimal residual disease (36). The compelling results obtained with nanobodies-based approaches support the broader application of nanobodies in various CAR-based immunotherapies, offering a viable treatment for diseases with limited therapeutic alternatives. Similarly, it is reasonable to expect that VHH-based CAR-NK cell therapy would generate a deep and durable response in patients with MM.

In addition to CAR target recognition, extensive research has focused on optimizing the intracellular signaling moiety, which has been intensively examined to maximize immunomodulatory signals mediated by co-stimulatory molecules, often derived from the CD28 or TNFR gene family (**Table 1**). The core stimulatory molecule, CD3ζ, is an integral component of every CAR construct due to its essential role in lymphocyte activation and promoting CD16-mediated antibody-dependent cell-mediated cytotoxicity (ADCC). Costimulatory proteins from the CD28 family, such as CD28 and ICOS, transmit intracellular signals via the phosphatidylinositol 3-kinase (PI3K)-Akt pathway, while 4-1BB, OX40, and CD27 of the TNFR family rely on TRAF proteins for signal transduction (59). CD28 and 4-1BB are commonly employed molecules to augment activation signals and redirect effector cell cytotoxicity towards cancer cells. CD28-integrated CARs trigger faster T cell activation, proliferation, and cytolysis but shorter persistence. Conversely, 4-1BB-based CARs enhance T-cell endurance by promoting oxidative metabolism and mitobiogenesis, resulting in slower effector responses (60). Activation through CD3ζ and 4-1BB allows genetically modified NK cells to overcome killer immunoglobulin-like receptors (KIR)-mediated inhibitory responses in B cell malignancies (61). Furthermore, 4-1BB signaling activates the PI3K and MEK ½ pathways to enhance cell cycle progression, boosting the potency and persistence of CAR-expressing effector cells to a larger extent than CD28 (62). 2B4, a member of the SLAM family, is a costimulatory molecule specific to cytotoxic lymphocytes. Signal transduction through 2B4 requires the recruitment of SLAM-associated protein (SAP) for NK cell activation (63).

**Table 1:** CAR-NK platforms to target liquid tumors.

Type of Cancer	Target Antigen	Intracellular Domain	References
MM	CD138	CD3ζ	(37)
MM	CS1	CD28-CD3ζ	(38)
MM	NKG2D	4-1BB-CD3ζ	(39)
MM	BCMA	2B4-CD3ζ	(40, 41)
MM	BCMA	unknown	(42)
MM	BCMA GPRC5D	unknown	(31)
MM	GPRC5D CD38	unknown	(43)
MM	BCMA	unknown	(44)
T-ALL	CD5	CD28-4-1BB-CD3ζ	(45)
B cell lymphoma	CD19	CD28-4-1BB-CD3ζ	(46)
B-ALL	CD19	4-1BB-CD3ζ	(47)
B-ALL	CD19	CD28-CD3ζ	(48)
B-cell leukemia	CD19	CD28-CD3ζ 4-1BB-CD3ζ	(49)
B-ALL	CD19	4-1BB-CD3ζ	(50)
B-ALL	CD19	CD28-CD3ζ	(51)
B-lineage leukemia	CD19	CD3ζ	(52)
B-ALL	FLT3	CD28-CD3ζ	(53)
CLL	CD19	CD28-CD3ζ-IL15	(54)
B-NHL	CD20	4-1BB-CD3ζ	(55)
Burkitt lymphoma	CD20	4-1BB-CD3ζ	(56)
Burkitt lymphoma	CD38	CD28-4-1BB-CD3ζ	(57)
NHL	CD4	CD28-4-1BB-CD3ζ	(58)

CD28/4-1BB/2B4/CD3ζ CAR-NK cells have shown potent anti-MM response in various *in vitro* and *in vivo* settings (23, 31, 32, 42). For example, CD3ζ, and 4-1BB have been incorporated into CAR-NKG2D to promote the antitumor activity of NK cells (39, 64). 2B4-based CAR-NK cells were found to elicit potent effector response and durable anti-MM activity, supporting the translation of FT576 (BCMA CAR-NK) (44). The FDA-approved Abecma and Carvykti for R/R MM contain CD3ζ and 4-1BB (65, 66). Overall, this indicates each malignancy requires a specific incorporation of CD3ζ backbone and co-stimulatory protein(s) in CAR constructs to each type of effector cells, and the various combination of CD28/4-1BB/2B4/CD3ζ is preferably used as intracellular domains of CAR-NK cell therapy.

As cytotoxicity of NK cells relies on the balance between inhibitory and activating receptors, disrupting such balance would enable the innate lymphocytes to be more aggressive toward malignant cells. However, each subset of NK cells displays a distinct phenotype. Depletion of NKG2A, a constitutively expressed inhibitory receptor, was demonstrated to be a potential strategy to enhance the cytotoxicity of memory-like NK cells but not conventional NK cells (67). When the receptor is suppressed, NKG2A-mediated cytotoxic functions are unleased, leading to better recognizing and cytotoxic responses. NKG2A-deficient NK cells display enhanced IFN-γ production, a crucial proinflammatory cytokine in activating cellular immunity and stimulating antitumor responses (68, 69). Blocking NKG2A expression or its interaction with HLA-E has shown positive outcomes in preclinical studies and early-phase clinical trials as a strategy for enhancing anti-tumor immune responses (67). This suggests multidimensional and thorough analyses are needed to elucidate the physiological role of those receptors, thereby precisely targeting and exploiting therapeutic effects in different cancer settings.

An advanced CAR-based strategy involves reducing fratricide (cells to kill their siblings) of bioengineered immune cells without compromising CAR-mediated antitumor responses, in which both inhibitory CAR (iCAR) and activating CAR (aCAR) are incorporated into NK cells (70). Trogocytosis is a rapid process of surface protein uptake cells (71). Both CAR19-transduced T cells and NK cells undergo trogocytosis in cancer settings, but the antigen-uptake action is less dramatic in the former. A positive correlation between trogocytic antigen acquisition and reduction of CD19 mediated through immunologic synapse formation is observed (70). This study proves that mitigating the fratricide of NK cells is feasible without impairing key anti-tumor activities through implementing a novel dual CAR system in which iCAR-mediated responses overdrive a CAR-induced immunomodulatory consequence. Logic-gated strategies can be applied but not limited to NK cells to further enhance the anti-MM activity of CAR-modified immune cells. Overall, elucidating the fundamental roles of inhibitory and activating receptors of NK cells in the context of cell-cell interaction and cellular homeostasis would critically drive the development of sustained NK cell-based immunotherapy.

The safety concerns associated with adoptive cellular therapy, such as an incomplete understanding of CAR-expressing cell behavior, uncontrolled immune responses, and the risk of lymphoma caused by infected allogeneic lymphocytes after infusion, are critical areas for advancing translational research. One strategy to address these concerns is incorporating an inducible suicide switch, such as the inducible caspase-9 (iCasp9) system, into CAR-expressing cells. This allows swift elimination of the cells through activation of the suicide switch using a specific chemical inducer of dimerization (54). Indeed, iCasp9 has been integrated into CAR19 to ensure the safety of CAR-NK cell therapy in humans (13).

An out-of-the-box strategy has been tested to produce CAR-expressing cells to obviate some medical complications associated with allogeneic transfusion. For heavily treated patients, the number of effector cells is frequently insufficient for conducting adoptive immune cell therapy. This resolves the need for an innovative approach that enables limited effector cells to expand, acquire transgenes, and exhibit newly desirable functionalities. Unlike most of the traditional methods to generate CAR-T *in vitro*, CD19-targeted CAR T cells are generated *in vivo* by the use of T cell-specific lentiviral vectors (LVs) CD4 and/or CD8-LV indicating an unconventional way to produce CAR-expressing immune cells (72–74). Macrophages emerge as the potential barrier for gene transfer, engineering the vector surface to reduce the immunogenicity of the carriers overcomes the macrophage obstacle resulting in more efficient gene transduction (74). A similar approach can be used to generate CAR-NK in situ. For example, envelope proteins are first modified to suit the delivery to NK cells. NK cell specificity can be obtained by fusing the envelop proteins with a NK-specific scFv (for example, NKG2D scFv). Although promising, *in vivo* generation of CAR-expressing cells using viral or non-viral particles requires thorough engineering and preclinical testing (i.e., antitumor efficacy, cytokine profile, yield of gene transfer, biodistribution, immunogenicity, and off-target transfer) before translating to human use.

#### 2.2.2 CRISPR-based editing to improve NK cell effector response

The translational development of genetically modified NK cells NK has been hindered by the intrinsic susceptibility of NK cells to exotic genetic materials. Advances in genome editing and viral-based delivery resolve challenges associated with editing and CAR transduction into NK cells, respectively. Efficient CRISPR platforms have been developed to genetically manipulate NK cells for immunotherapy (75–77).

Traditional viral vectors face several concerns, including safety, scalability, and transduction efficiency. To overcome these challenges, more effective viral vector-based transfection systems, such as Baboon envelope pseudotyped lentiviral vectors, and non-viral gene delivery methods like electroporation, transposon systems, and nanoparticles, have been established and reported elsewhere (78–81). Thus, the therapeutic potency of adoptive NK cells can be enhanced by CRISPR editing. In this section, we focus on CRISPR platform to edit the NK cell genome for therapeutic purposes.

The combination of genome editing and CAR technology presents a novel approach to enhance the metabolic fitness and the antitumor activity of CAR-expressing cells. CRISPR-Cas9 enables targeted insertion of the CAR construct into a specific genomic locus, minimizing the risk of insertional mutagenesis and off-target effects. As various tumor intrinsic and extrinsic resistance mechanisms can cripple NK cell function (82), CRISPR/Cas9-based precision genome editing serves as a potent tool to overcome functional exhaustion, limited migration, and impaired persistence. Using the Cas9 platform, the CD38 knockout rate of peripheral blood NK cells was relatively high (>80%). When whole genome sequencing was performed to identify mutations that may result from off-target editing, only 4 off-target genes with potentially high-impact mutations (startloss, stopgain, and frameshift) were found in target NK cells, demonstrating the high efficacy of the CRISPR/Cas9 platform (83). The generated CD38^-/-^ NK cells resist to daratumumab-induced conjugation and fratricide. These CD38^-/-^ NK cells also demonstrate enhanced ADCC activity against MM cell lines with low CD38 expression and MM cells obtained from a non-responsive daratumumab (DARA)-treated patient, compared to CD38 wild-type NK cells. The deletion of CD38 resulted in reprogramming key metabolic events, such as increased mitochondrial respiration and oxidative phosphorylation, likely contributing to their enhanced functional longevity against MM (83). These findings suggest CD38-depleted NK cells could benefit DARA-treated patients with residual low CD38-expressing MM cells. Another CRISPR/Cas9 engineering application in the CAR-NK approach involves targeting the CIS protein (encoded by *CISH*), a component of the IL-15 signaling axis, to enhance *in vivo* persistence and cytotoxic functionality (84). The *CISH* knockout rate was also encouraging (>80%), while the CAR transduction efficiency and cell viability reached >90%. The CAR expression was stable over time in transduced cells. To mitigate off-target editing events mediated by CRISPR-Cas9 RNP complexes, genome-wide off-target effects of CISH gRNAs are assessed using guide-seq and rhAmpSeq technologies in HEK293 cells. Although Cas9 exhibited a low frequency of off-target events with the utilized CRISPR RNAs, the off-target events were further reduced to less than 0.5% by employing a high-fidelity Cas9 protein (84). This IL-15 signaling-targeted approach enhances NK cell-mediated antitumor responses, leading to the complete elimination of lymphoma grafts.

To further boost ADCC, suppressing NK cell inhibitory signaling using a highly efficient CRISPR/Cas9 platform was also reported, in which the editing efficiency achieved >80% with >98% transfection efficiency and 90% cell viability (76). Knockout of ADAM17 (a negative regulator of ADCC) or CRISPR knock-in of a non-cleavable CD16a variant increased ADCC. However, the capacity of tumor cells to suppress NK cell-mediated ADCC via inducing inhibitory signals, including PD-L1 and PD-1 interaction, makes targeting PD-1 on NK cells a viable approach. Indeed, knockout of PD-1 significantly enhanced NK cell-mediated ADCC to cancer cells (76). Collectively, these data indicate the clinical translation potential of the CRISPR/Cas9 platform to improve the clinical efficacy of adoptive NK cell therapy.

Although powerful, CRISPR/Cas9 technology requires double-stranded breaks for editing properly. This may cause genome instability. Base editing has been developed with enormous potential in treating genetic diseases, especially point mutation-associated disorders. Unlike other DNA manipulation methods, base editing enables precise and irreversible conversion of DNA base without causing DNA breaks, thus preserving chromosomal stability (85, 86). Promising preclinical results have paved the way for base-edited CAR-T clinical trials (87, 88). For the first time, the CRISPR-guided base editing approach was proven effective in human applications to treat relapsed CD7-expressing T-ALL (89). Base editing has also been applied to improve effector functions of NK cells with high editing rates and knockout protein levels (90). Targeting multiple genes (i.e., ARH, CISH, KLRG1, TIGIT, KLRC1, PDCD1, and CD16A) that inhibit NK cell effector function did not affect the base editing efficiency (95%) in NK cells, while deletion of CD16A significantly increased NK cell-mediated ADCC (90). As the safety and feasibility of this editing technology are demonstrated in T cells, NK cell-based immunotherapy would likely gain translational advancement for clinical use by titrated and timed depleting inhibitory signals or enhancing metabolic fitness.

### 2.3 Metabolic engineering to increase antitumor functionality and persistence of NK cells

**Figure 2:**
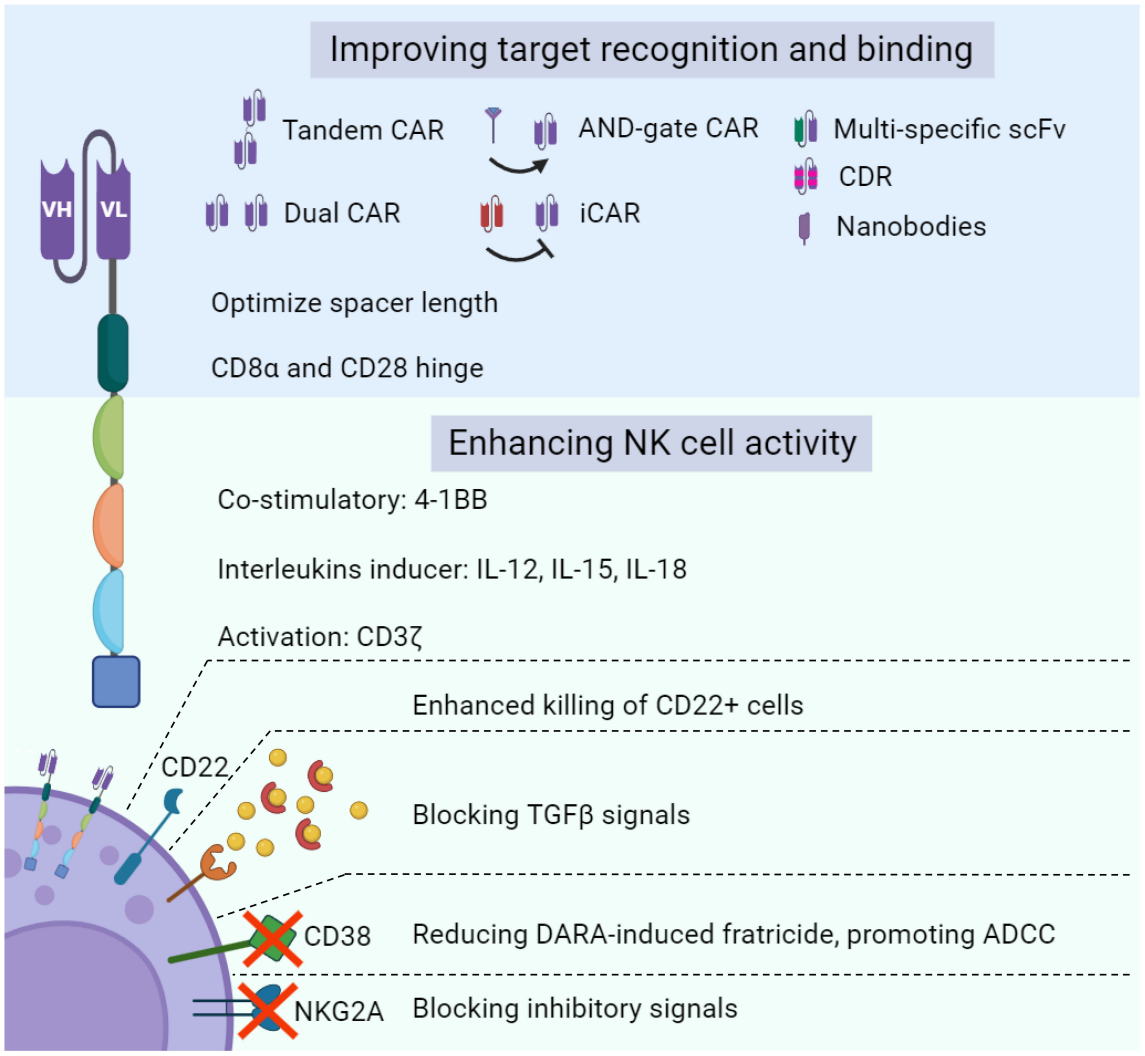
Bioengineering approaches to enhance NK cell function. Legend 2: various molecular approaches can be deployed to empower NK cells with novel functionalities and enhance NK cell-based tumor-killing capacity. Optimizing target recognition and binding of CAR-NK therapy through targeting multiple cancer antigens and taking advantage of inhibitory KIR signals are major strategies to boost NK cell cytotoxicity toward cancer cells. In addition, negative regulators of NK cell metabolism are another primary target to improve antitumor activity.

Modulating NK cell metabolism has been a strategic approach to enhance the therapeutic efficacy of adoptive NK cell therapy (54, 91–93). Cytokines (i.e., IL-12, IL-15, IL-18, TGF-β) and other vital metabolic regulators of the innate lymphocytes are often manipulated (**Figure 2**).

IL-12, a pro-inflammatory cytokine, plays a central role in regulating innate and adaptive immunity and essentially involves in the generation of memory-like NK cells (94, 95). Integrating IL-12 into CAR-T exodomain reprograms cellular functional capacity by converting CD8^+^ T cells to multi-functional NK-like cells. Intriguingly, simultaneous signaling coordinated by the CD28-ζ CAR and IL12 is required for potent antitumor activity (91). Similarly, MUC16-targeted CD28-ζ CAR T cells failed to reduce xenograft tumors, but the addition of IL12 endowed the CAR-T cells with effective tumor control (96). Given that IL-12 plays a role in the secretion of IFN-γ and control of NK cell proliferation and cytotoxicity, incorporating IL-12 into CD28-ζ CAR construct would be a promising strategy to enhance NK cell antitumor response.

IL-18 is a pleiotropic cytokine that activates NK and cytotoxic T cells (97). IL-18 augments IFN-γ production in NK cells and CD16-mediated cytolytic activity (98). This cytokine also strongly promotes NK cell proliferation and confers NK cells an APC-like phenotype to eliminate tumor cells effectively (99). Due to its essential roles in immune cells’ development and antitumor activity, IL-18 is incorporated into CAR-T constructs to promote T cells’ proliferation, persistence, and antitumor activity (93, 100). Similar to IL-12, how IL-18 incorporation modulates effector functions, expansion, and persistence of adoptive NK cells remains encouraged to be investigated.

IL-15 is one of the best-studied signaling molecules of CAR technology with somewhat contradictory data (13, 54, 101, 102). In a preclinical study, 2B4.ζ CAR but not 4-1BB CAR-NK cells displayed improved AML-killing efficacy. The secreted IL-15 augmented CAR-NK cell expansion and anti-AML cytotoxicity but caused systemic toxicity, which was attributed to the dramatic proliferation of NK cells associated with high circulating IL-15 and other proinflammatory cytokines levels causing early death in one of two used mice models (101). In CAR-NK therapy, IL-15 is the only interleukin whose presence is proven to contribute to clinical efficacy (13). Moreover, IL-15 significantly contributes to overcoming the loss of metabolic fitness, a primary drug-resistance mechanism in CAR-NK cell therapy (103). These findings set the notion for targeting the cytokine signaling axis to enhance the therapeutic effects of adoptive NK cell transfer.

One such translational approach is the knockout of *CISH*, a critical negative regulator of IL-15 signaling. Ablation of the CIS checkpoint lifted the restraint on IL-15 signaling, resulting in increased basal glycolysis, glycolytic capacity, maximal mitochondrial respiration, and ATP-linked respiration (104). As the role of IL-15 in NK cell metabolism is regulated by mTOR activation, inhibition of mTORC1 completely neutralized glycolysis and oxidative phosphorylation levels in the CIS-deficient iPSC-derived NK (iNK) cells compared with wild-type, demonstrating the mTOR pathway mediates the improved metabolic fitness and enhanced effector function. Rather than only targeting cancer cell-intrinsic factors, reprogramming effector cell metabolism via inhibiting intracellular immune checkpoints is a notable strategy to complement existing advanced adoptive immunotherapies.

TGF-β is an immune checkpoint of the TME and can suppress the anti-tumor activity of NK cells in the TME. Upregulation of TGF-β is associated with reduced granule exocytosis, tumor-killing activity, and IFN-γ secretion of NK cells by down-regulating their activating receptors, including NKG2D, NKp30, and DNAM-1 (105). TGF-β further impairs IFN-γ and ADCC of NK cells by inhibiting CD16 (106). Given such critical roles, blocking TGF-β in combination with a tumor target recognition platform (i.e., CAR technology) would further promote NK cell metabolic and antitumor responses. Indeed, TGF-β was intervened using small molecule inhibitors (GC1008, LY3022859) to reduce tumor burden and metastasis (107). An interleukin (IL)-15 superagonist/IL-15 receptor α fusion complex (IL-15SA/IL-15RA) functions as an inhibitor of TGF-β1 signaling to inhibit Smad2/3-induced transcription, resulting in the rescue of NK cell cytotoxic function (108).

Genetic modification of the TGF-β receptor is an alternative to exert activating signals instead of TGF-β-mediated inhibitory responses in TGF-β-secreting malignancies. Indeed, imprinting a mutant TGF-β dominant-negative to NK-specific activating domains renders the modified TGF-β receptor-expressing NK cells potent cytotoxicity against neuroblastoma, conferring significant protection to mice (109).

Glycoengineering has been developed to complement genetic engineering to augment immune cell-mediated antitumor response. CD22-targeted glycoengineered NK cells were generated using modified sialic acid derivatives, MPB-sia 1 and BPC-sia 2. The glycoengineered NK-92 cells showed enhanced binding affinity and cytotoxicity against CD22-positive lymphoma cells, effectively protecting B-cell lymphoma-challenged mice (110). As the expression of target protein in the chemically engineered cells is transient, this novel and simple platform would find a more prominent application niche in enhancing the homing and cytotoxic activity of CAR-expressing immune cells.

Collectively, these convincing preclinical results strongly support the premise of exploring and targeting key negative metabolic modulators of immune cells to overcome the TME and improve patient outcomes.

### 2.4 CAR-NK cell vs. CAR-T cell therapy in multiple myeloma

Advantages of adoptive T cells over NK cells include the potent cytotoxicity, specificity, and ease of genetic manipulation, while the presence of TCR in T cells essentially hinders their allogeneic application. NK cells, on the other hand, are entitled to off-the-shelf features (**Table 2**). The integration of CAR has offered NK cells unique functionalities that are not available in primary NK cells, including antigen specificity, tumor trafficking, better persistence, and expansion.

**Table 2:** CAR-NK vs CAR-T cell therapy.

Cellular therapy	Target antigens	Advantages	Disadvantages
**CAR-T**	Surface TAAs	- Potent cytotoxicity - Durable clinical response - Single infusion - Long-term benefit due to ‘‘living drug” nature	- High relapse rate - Mainly autologous - CRS - Neurotoxicity - Poor tumor trafficking capacity - High cost
**CAR-NK**	Surface TAAs	- High safety profile with no CRS, neurotoxicity, and GvHD in an allogenic setting - Inherent antitumor functionality - Off-the-shelf manufacturing	- Resistance to genetic engineering - Poor tumor trafficking capacity - Requiring more clinically demonstrated efficacy

Development of the NY-ESO-1-targeted TCR-T therapy in MM was discontinued after showing good clinical response (111). In contrast, BCMA CAR-T cell products have already earned FDA approval in R/R MM. The hematological malignancy still returns to CAR-T-cell-infused patients after 12-13 months, albeit with a high response rate (10, 112). This fuels the optimization of the current CAR-T platform as well as identifying better alternatives. CAR-NK cell therapies in MM are in the very early development stage with actively ongoing research.

Antigen escape and the hostile TME are determined as the significant causes of the therapeutic resistance of MM in response to CAR-T immunotherapeutic treatment. To tackle antigen downregulation, CAR-T cell targeting other MM-associated epitopes (i.e. CD138, CD38, CD19, GPRC5D, SLAMF7/CS1, APRIL, TACI, CD229, CD56, MUC1, NKG2D ligands, integrin β7, Kappa light chain, FcRH5, CCR10 and CD44v6) rather than BCMA alone have exhibited encouraging results (113). Likewise, CAR-NK cells target the same MM-associated antigens, including CD38, CD138, BCMA, and SLAMF7/CS1, and also have shown preclinical activity (6, 114).

A NK-specific BCMA-targeted CAR constructed from 4 transgenes and intracellular signaling modalities derived from NKG2D, 2B4, and CD3ζ has shown translational results with potent antitumor activity against MM in various mice models (40). The integration of non-cleavable CD16 (hnCD16) to the cassette and knockout of CD38 substantially increased ADCC-mediated antitumor response and mitigated fratricide, respectively, while the expression of membrane-bound IL-15 fusion promotes iNK cell metabolic fitness (40). To enhance therapeutic efficacy, the tumor necrosis factor-related apoptosis-inducing ligand (sTRAIL) is integrated into the CAR-BCMA construct to induce apoptosis in TRAIL-R upregulated cancer cells. The notion of more significant antitumor responses derived from the multimodality approach is also supported by the preclinical study’s findings, in which a dramatic synergistic cell lysis effect toward MM was obtained by using a combination of the engineered NK-92 cells therapy combined with targeted inhibitors (Bortezomib or γ-secretase inhibitors) (42). Given the therapeutic relevance of targeting BCMA in MM, several groups have generated dual CAR targeting BCMA and other antigens of interest, such as MICA/MICB or GPRC5D, to address the efficacy limitations of CAR-T (31–33). These dual-targeting CARs also empowered NK cells with potent and durable antitumor response against MM cell lines that highly or poorly express the antigenic proteins.

Poor infiltration of effector cells to tumor sites is one limitation of cellular immunotherapy (115). Ex vivo manipulation of lymphocytes downregulates CXCR4, a crucial homing molecule for lymphocytic cell trafficking to the bone marrow by binding to CXCL12/SDF-1α (116). Enforced expression of the chemoattractant protein may improve lymphocyte retention in bone marrow and disease control. Indeed, increased migration toward bone marrow niche-expressing chemokine CXCL12/SDF-1α of CXCR4-modified NK cells *in vitro* and a surged infiltration in bone marrow compartments *in vivo* were observed. Interestingly, intravenous injection of the CXCR4 and anti-BCMA CAR-NK cells significantly decreased tumor burden and prolonged survival of MM-grafted mice (117).

SLAMF7/CS-1, a highly expressed protein in MM cells but low in most immune cells and healthy tissues, may promote MM cell adhesion, clonogenic growth, and tumorigenicity, making them a rational target of MM (38, 118, 119). CS-1 was manipulated to improve therapeutic efficacy as well as prevent fratricide in CAR-T cells (119); however, deletion of CS-1 only protected the CAR-T cells from fratricide but did not enhance tumor control compared to CS-1-expressing CAR-T cells. In the CAR-NK setting, the function of CS1-scFv is amplified through a 2nd gen CAR construct containing an intracellular signaling domain of CD28-CD3ζ. CS1-specific CAR-NK cells exhibited potent antitumor responses against MM primary cells and cell lines, and adoptive transfer of the CAR-NK cells suppresses MM proliferation in a xenograft model. The degree of therapeutic effect of ablating CS-1 expression in CS-1-CAR-NK cells remains uninvestigated.

Another therapeutic target antigen for MM is CD138. This protein is often overexpressed on the MM cell surface, and blocking CD138 sensitizes myeloma cells to chemotherapy (120).

CAR138 T cells using NK-92-derived anti-CD138 scFv, CD8α, 4-1BB, and CD3ζ are safe, tolerable, and have first shown a glimpse of clinical effect (121). NK92 cells were modified to express CD138-targeted CAR in both *in vitro* and *in vivo* models (37). This 1st gen CAR is constructed from an anti-CD138 scFv sequence fused with CD3ζ to successfully render NK92 cells with enhanced antitumor activity toward CD138-expressing MM cells, surprisingly unaffected by irradiation. This preclinical study leaves a plenty of room for improvement as other CAR components, such as additional costimulatory domain, homing factor, or autologous interleukin, were not incorporated in the original synthetic construct. However, targeting CD138 is problematic due to the reduced expression of CD138 on primary MM cells in patients with relapsed/progressive disease (122). This may be the reason why CD138 has not been further clinically tested in MM using cellular immunotherapy.

Other overexpressed antigens on viral infected or aberrant cells are NKG2DLs. The epitopes are not usually expressed in healthy tissues, thus can be a feasible target. By another approach, autologous NK cells are taken out of MM patients and then undergo activation and expansion (AE) before being imprinted with a novel NKG2DL-specific CAR containing the extracellular domain of the NKG2D receptor (39). The CAR-NK platform showed translational implications accompanied by cytotoxicity toward MM cells but not normal cells and administration of MM-engrafted mice with the CAR-NK cells resulted in effective control of MM growth. Although the exact CAR-NKAE-mediated killing mechanism is unknown, the CAR-expressing NKAE cells exhibited increased expression of genes involved in cell activation, migration, exocytosis, and immune effector process.

Efforts in the therapeutic development of CAR-NK cell therapy for MM can also shift to other surface antigens such as CD229, integrin β7, CD70, and CD126 due to their prognostic value, and role in plasma cell biology.

## 3 Adoptive NK cell manufacturing: retaining effector functions after freeze-thaw cycles

Fresh CAR-based cellular immunotherapy such as CAR-T and CAR-NK cell products are required to infuse to patients, complicating and limiting the accessibility of adoptive immune cell therapy. An off-the-shelf approach is ideal to replace current adaptive immune cell therapy platform to timely provide banked cells to patients in need, lower cost of manufacturing and handling risk, and to conduct multi-site studies. As a result, retaining effector functions of adoptive NK cells after freeze-thaw cycles is critical.

To meet the unmet demand for immune cell-based anticancer therapy and to reduce the risk of human-related factors, it is necessary to automatically produce a large number of effector cells for downstream manufacturing and processing. The prevalent method involves growing the separated NK cells in a stationary culture and moving them to rock bioreactors, efficiently supporting and maintaining large cell populations (123). A rocking motion bioreactor enables gentle mixing of the cell culture, promoting homogeneity and preventing cell settling. This helps maintain NK cell viability while supporting efficient nutrient and gas exchange, providing a controlled environment for cell growth and optimizing culture conditions, including temperature, pH, gas exchange, and nutrient supply (124). The feasibility of a decentralized manufacturing process for CAR-T cells using an automated closed system in a developing country setting positions the feasibility of that high-volume production of CAR-expressing cells for clinical use (125–127).

Optimizing storage and handling conditions for CAR-NK cells is needed to preserve cytotoxic and effector functions of banked cells (128, 129). Several factors are known to influence cryopreserved cells’ post-thaw recovery and functionality, including the selection of cryoprotective agents and the cooling rate during freezing, storage, and thawing conditions. The rate at which the temperature decreases during freezing significantly affects post-thaw survival, emphasizing the importance of controlled-rate freezing for optimal outcomes. Proper storage conditions, such as temperatures below -150°C, are essential for maintaining cell stability (130).

Next, cryopreservation of NK cell-based therapies is challenging because cryopreserved effector cells often exhibit poor post-thaw viability, loss of motility, reduced expression of activating receptors, and functionality compared to fresh cells (131) (132–134). The reduced proliferation and cytotoxicity post-thaw can negatively impact their therapeutic efficacy, leading many clinical trials to opt for fresh NK cells despite the logistical difficulties associated with maintaining fresh cells (135, 136). Efforts have been made to optimize cryopreservation methods and develop alternative preservation techniques to address the challenges in cryopreserving innate and adaptive lymphocytes.

Adding an optimal amount of osmolytes enables cells and tissues to endure unfavorable conditions and stresses. The universal use of dimethyl sulfoxide (DMSO) has been challenged by its cytotoxicity and adverse events upon administration of thawed cells to patients. Prolonged exposure time to DMSO directly impacts cell viability and functionality, highlighting the demand for DMSO-free cryoprotectants (137). Each osmolyte has a unique role in maintaining cell viability. For example, sugars support the stabilization of the cell membrane, glycerol helps to stabilize cellular proteins, while amino acids prevent sugar precipitation. Indeed, a combination of the aforementioned osmolytes improved stabilizing Jurkat cells and mesenchymal stromal cells during freezing (138). Furthermore, higher glycerol concentrations increased post-thaw cell recovery. Similarly, DMSO-free media may enhance the preservation of dendritic cells by preventing DMSO-induced cytoskeleton depolymerization, thus improving post-thaw cell function (139). New cryopreservation and thawing technologies, such as LN2-free controlled-rate freezers and dry thawing devices, have been developed to acquire more consistent and standardized methods for freezing and thawing, reducing contamination risks and improving cell recovery (140). A simple and effective particle-based method to expand and cryopreserve NK cells is stimulating the cells with PM21 particles (prepared from K562-mb21-41BBL cells) (141, 142). Based on several lines of evidence, it is hinted that adding PM21 particles preserve cytotoxicity and expression of activating receptors of the stimulated NK cells compared to fresh NK cells. This suggests that osmolytes jointly function to improve post-thaw recovery by limiting the crystallization of intracellular water, thus in-depth understanding of how osmolytes act in concert is valuable to enhance cell viability.

Functional NK cells can be obtained from various sources, including the NK-92 cell line, peripheral blood cells, umbilical cord blood (CB), and iPSCs. The generation of iPSC-derived effector cells, including T cells, NK cells, macrophages and neutrophils, represents a significant progress in improving the accessibility of adoptive immune cell therapy.

**Figure 3:**
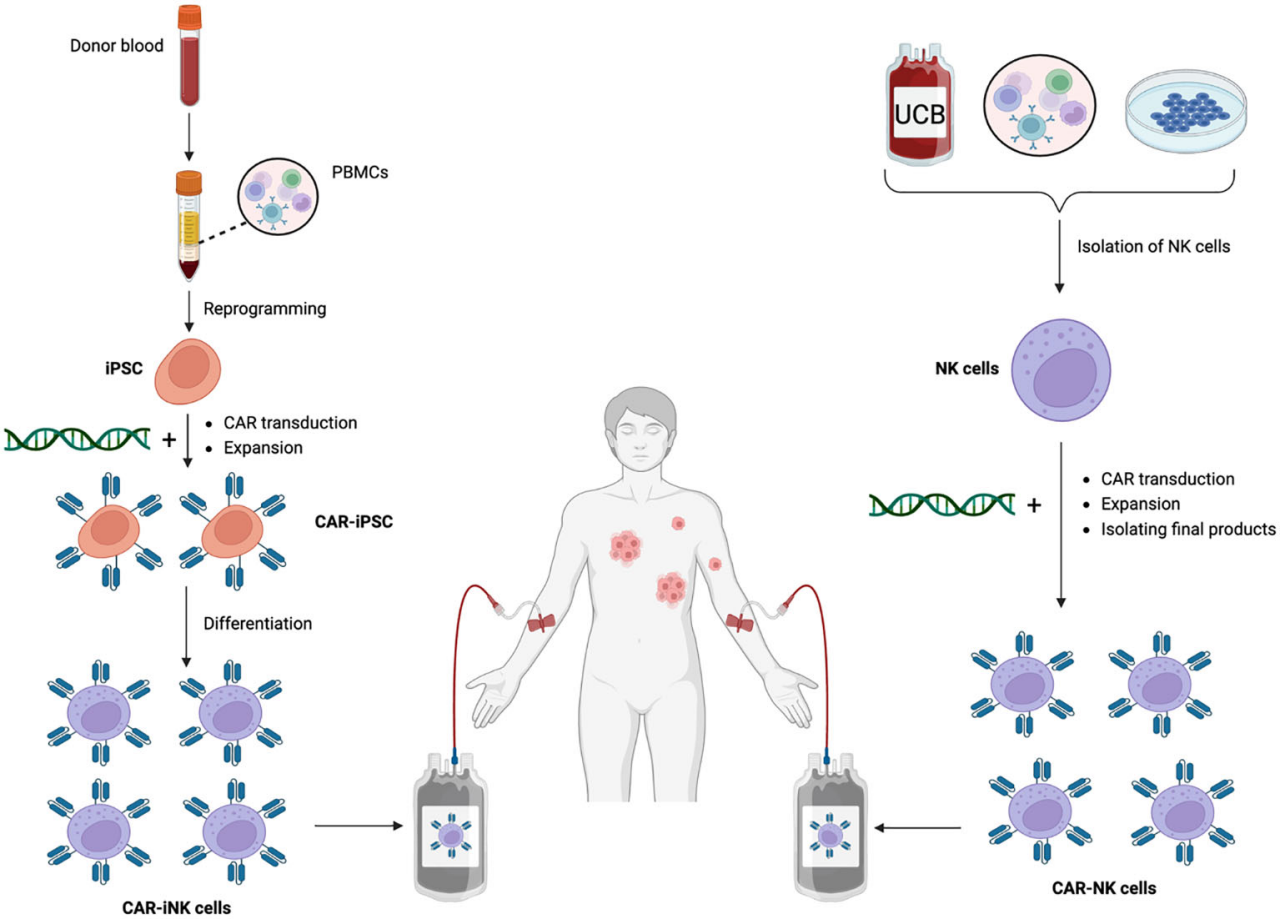
CAR-iNK vs. CAR-NK cell therapy. Legend 3: CAR-iNK vs. CAR-NK cell therapy. CAR-modified NK cells can be manufactured from different sources, including whole blood, UCB, and NK cell lines. For CAR-iNK cells, whole blood from donors is collected and then subjected to gradient density to collect the buffy coat that contains PBMCs. Next, PBMCs undergo reprogramming through multiple selection steps to become iPSCs which are transduced with CAR constructs and expand to a desirable number. These CAR-iPSCs are then differentiated into homogenous, ready-to-infuse CAR-iNK cells. NK cells are first isolated for CAR-NK cells generated from more traditional sources. Next, synthetic constructs are integrated into the NK cells, followed by expansion, purification, and quality control before infusing them into patients.

Large-scale off-the-shelf iNK cell production has been established (143, 144), in which transcriptional profiling shows a considerable similarity between iNK and peripheral blood NK cells. Importantly, innate and modified receptor-mediated antitumor activity are also retained after a freeze-thaw cycle. This innovative platform overcomes the limitations associated with traditional NK cell sources, such as inconsistency and finite cell quantities, by providing a consistent and virtually limitless supply of functional NK cells. CAR-iNK cells have shown considerably encouraging results in preclinical studies, and their use can simplify the manufacturing process and enhance the consistency of the final product (145). Given these engineered homogenous NK cells can be produced in advance and stored for use as needed, this makes them a suitable off-the-shelf product to reduce cost and waiting time for patient, promoting treatment accessibility to a larger patient population (41). Moreover, this iPSC-derived cell therapy synergizes with anti-PD-1, bolstering antitumor responses via recruiting and corporating with T cells (143). iNK cell-based therapies have entered clinical trials (NCT03841110, NCT04023071) as monotherapy or in combination with ICIs for cancer treatment with high expectations. A direct comparison between CAR-NK and CAR-iNK cell therapy is described in **Figure 3**. Given the noticeable limitations of irradiated NK-92 cells and CB/PB-derived NK cells, it is likely that iPSC-NK cells would triumph in adoptive NK cell therapy.

In another context, Fate Therapeutics has developed a cryopreservation method that enables high-cell density fill (HD-fill) doses of engineered iNK cells to be administered to patients (146). Although the formulation was not revealed, this platform demonstrated a comparable recovery and viability between standard and HD-fill densities, accompanied by retained transgene expression and cytotoxicity. Moreover, HD-fill bioengineered iNK cells can be preserved in liquid nitrogen and deep freezers (-80°C), which are available at most hospitals worldwide. These findings may pave the way for the implementation of advances in cryopreservation in clinical trials, but it would probably take several more years to have obtained credible cryopreservation methods for human use.

## 4 Adoptive NK cell therapy in clinical trials

Adoptive NK cell therapy is growing at an exponential pace (**Table 3**). Before 2019 there were only 4 CAR-NK clinical trials, and after 2019 the number soared to more than 40. Half of the CAR-NK trials are in phase I/II and actively recruiting patients, 14 in the early phase I, two completed trials (NCT03056339 and NCT02944162), and one terminated. Clinical trials involving CD7, CD19, GPRC5D and/or BCMA-targeted CAR-T cells, and CD19-targeted CAR-NK cells have delivered encouraging results in treating hematological malignancies (89, 147). Among the CAR-NK constructs, CD19 remains the preferred target, with 20 trials conducted on patients with B cell lymphoma.

**Table 3:** CAR-NK clinical trials.

Phase	Study period	Target antigens, CAR construct & transfection method	Sources of NK cells	Cancer type	NCT number
Phase I-II	Jun 2017 - Mar 2023	CD19-CD28-zeta-2A-iCasp9-IL15 Retroviral vector	CB	B-lymphoid malignancies (ALL, CLL, NHL)	NCT03056339
Phase II	Mar 2020 – May 2039	CD19-NKG2D-2B4- CD3ζ-IL-15/R-hnCD16 Lentiviral vector	iPSC	R/R BCLs and CLL	NCT04245722
Phase I	May 2021 – May 2024	Construct not disclosed Target CD19 Lentiviral vector	Haploidentical NK cells	B-cell NHL	NCT04887012
Phase I (Terminated)	Oct 2021 - Dec 2022	Construct not disclosed Target NKG2DL	CB	R/R AML	NCT05247957
Phase I	Sep 2022 – Sep 2025	Construct not disclosed Target CD19	CB	B-cell NHL	NCT05472558
Early phase I	Jan 2023 – Dec 2024	Construct not disclosed Target CD19	N/A	R/R diffuse large BCL	NCT05673447
Phase I/IIa	Jun 2022 – May 2034	Claudin6 CAR-NK expressing IL7/CCL19 and/or scFvs against PD1/CTLA4/Lag3	PB	CLDN6+ advanced solid tumors	NCT05410717
Early phase I	Oct 2022 – Oct 2024	Construct not disclosed Target CD123	N/A	R/R AML	NCT05574608
Early phase I	Mar 2023 – May 2025	Construct not disclosed Target CD19	N/A	R/R B-cell ALL, BCL, CLL	NCT05739227
Phase I	Dec 2021 - Jun 2024	Construct not disclosed Target NKG2DL	N/A	Refractory metastatic colorectal cancer	NCT05213195
Phase I	Jan 2023 – Nov 2023	Construct not disclosed Target NKG2D	NK-92 cell line	R/R solid tumors	NCT05528341
Phase I	Dec 2022 – Dec 2024	Construct not disclosed Target CD19	N/A	Adult R/R B-cell hematologic malignancies	NCT05645601
Early phase I	Nov 2020 – Nov 2022	Construct not disclosed Dual target CD33/CLL1	N/A	AML	NCT05215015
Not applicable	Mae 2023 – Sep 2024	Construct not disclosed Target NKG2D	N/A	Platinum-resistant recurrent ovarian cancer	NCT05776355
Early phase I	Dec 2021 – Dec 2022	Construct not disclosed Target 5T4	N/A	Advanced solid tumors	NCT05194709
Not applicable	Mar 2023 – Sep 2024	Construct not disclosed Target NKG2D	N/A	R/R AML	NCT05734898
Early phase I	Dec 2020 – Dec 2023	Construct not disclosed Target CD19	N/A	R/R NHL	NCT04639739
Early phase I	Mar 2019 – Nov 2021	Construct not disclosed Target CD22	N/A	R/R BCL	NCT03692767
Early phase I	Mar 2019 – Nov 2021	Construct not disclosed Target CD19	N/A	R/R BCL	NCT03690310
Phase I	Dec 2021 – Dec 2023	Construct not disclosed Target CD33	N/A	R/R AML	NCT05008575
Early phase I	Nov 2022 – Nov 2023	Construct not disclosed Target BCMA	N/A	R/R multiple myeloma	NCT05652530
Early phase I	Mar 2019 – Nov 2021	Construct not disclosed Target mesothelin	PB	Ovarian epithelial cancer	NCT03692637
Phase I	Jan 2018 – Dec 2019	Construct not disclosed Target NKG2DL	N/A	Metastatic solid tumors	NCT03415100
Phase I	Sep 2022 – Jun 2023	Construct not disclosed Target DLL3	N/A	Extensive stage small cell lung cancer	NCT05507593
Early phase I	Oct 2021 – Sep 2023	Construct not disclosed Target BCMA	UB and CB	R/R multiple myeloma	NCT05008536
Phase I Phase II	May 2019 – May 2022	Construct not disclosed Target ROBO1	N/A	Solid tumor	NCT03940820
Phase I Phase II	May 2019 – May 2022	Construct not disclosed Target BCMA	NK-92 cell line	R/R multiple myeloma	NCT03940833
Phase I	May 2022 – May 2024	Construct not disclosed Target CD19	N/A	ALL, CLL, NHL	NCT05410041
Phase II	Dec 2021 – Dec 2025	Construct not disclosed Target PD-L1	N/A	Recurrent/metastatic gastric or head and neck cancer	NCT04847466
Early phase I	Feb 2019 – Jan 2021	Construct not disclosed Dual-target CD19/CD22	N/A	R/R BCL	NCT03824964
Phase I	Apr 2021 – mar 2024	Construct not disclosed CD19-IL15 Retroviral vector	CB	ALL, CLL, NHL	NCT04796675
Phase I	Sep 2020 – Jul 2038	NKG2D-CD134 (OX40)- CD3ζ -IL-15	NK cells from haplo-matched donors	R/R AML Refractory myelodysplastic syndromes	NCT04623944
Phase I	Aug 2021 – Dec 2038	CD19-CD134 (OX40)-CD3ζ-IL-15	N/A	R/R NHL, CLL, B cell ALL	NCT05020678
Phase I	Jul 2022 – Nov 2022	T Construct not disclosed target CD19	N/A	R/R ALL	NCT05563545
Completed Phase I Phase II	Oct 2016 – Sep 2018	CD33-CD28-CD137 (4-1BB) Lentiviral vector	NK-92-MI cell line (expressing human IL-2)	R/R CD33+ AML	NCT02944162
Phase I	Dec 2022 – Dec 2025	CD19/CD70-IL-15 Lentiviral vector	CB	B cell NHL	NCT05667155
Phase I Phase II	Sep 2016 – Sep 2019	CD19-TCRζ-CD28-CD137 (4-1BB)	Allogenic NK-92 cell line	Various subsets of lymphoma	NCT02892695
Phase I Phase II	May 2019 – May 2022	Target ROBO1	N/A	Pancreatic cancer	NCT03941457
Phase I Phase II	May 2019 – May 2022	ROBO1 CAR-NK/T cells	N/A	ROBO1+ cancers	NCT03931720
Early phase I	Feb 2023 – Jun 2024	SZ011 CAR-NK	N/A	Advanced triple-negative breast cancer	NCT05686720
Phase I Phase II	Dec 2022 – Dec 2024	Anti-CD19 universal CAR-NK	N/A	B-cell lymphoblastic leukemia/lymphoma	NCT05654038
Early phase I	Dec 2018 – Jun 2024	Name: TABP EIC Target PSMA	N/A	Metastatic castration-resistant prostate cancer	NCT03692663
Phase I Phase II	Oct 2019 – Oct 2019	CD19-CD28-ζ-2A-iCasp9-IL15 CB-derived	CB	CD19+ B cell lymphomas	NCT03579927
Phase I	Dec 2022 – Dec 2025	Construct not disclosed Target CD33	Allogeneic NK cells from donors	R/R AML adult	NCT05665075
Phase I	Oct 2022 – Oct 2025	Construct not disclosed Target CD33	Allogeneic NK cells from donors	R/R AML adult	NCT05601466
Phase I	Nov 2021 – Dec 2024	Construct not disclosed Target CD19	NA	B cell ALL	NCT05379647
Phase I	Nov 2021 – Feb 2040	BCMA-158V CD16-IL-15/IL-15R fusion, CD38-knockout	iPSC	R/R multiple myeloma	NCT05182073
Phase I	Dec 2017 – Dec 2023	ErbB2-CD28-CD3ζ NK-92/5.28.z	NK-92 cell line	HER2+ glioblastoma	NCT03383978
Phase I Phase II	Nov 2022 – Nov 2023	Construct not disclosed CD70-IL15	CB	R/R hematological malignancies	NCT05092451
Phase I	Jan 2023 – Aug 2027	Construct not disclosed CD19-IL15	N/A	R/R CD19+ B-cell malignancies	NCT05336409

Early crucial data on 11 heavily pretreated R/R CLL or non-Hodgkin lymphoma patients showed objective responses (OR) in 8 patients, CR in 7 patients upon infusing with CB-derived CAR19 allogeneic NK cells (13). These results imply that CB-NK cells support CAR’s stable and efficient expression with promising preliminary efficacy and minimal safety concerns. NCT04245722 also targeted CD19 to treat BCLs and CLL (148). In two treatment regimens comprised of CAR-NK products and chemotherapy, clinical benefit was observed in 9 patients (5 from regimen A, and 4 from regimen B), of which 7 had CR. CAR-T immunotherapy is considered the last therapeutic option for patients with R/R tumors, leaving CAR-T-treated relapsed patients virtually incurable. Interestingly, 2/4 CAR-T-treated relapsed patients had CR. Although the sample size is small, this may indicate another milestone for CAR-NK therapy. However, detailed medical and physiological reports of enrolled patients have not been disclosed. Thus, deciphering the working mechanism responsible for the preliminary therapeutic responses (e.g., FT596 monotherapy, or rituximab combination) is required for further development of the CAR-NK therapy.

Another CAR-NK cellular product targeting CD19, NKX019, has shown encouraging clinical efficacy (149), in which the CR rate is up to 70% (7 out of 10 patients). Unlike CAR19 T cells, which primarily rely on CD19 antigen density for activity, NKX019 exhibited potent antitumor effects even when the target antigen density was drastically reduced. Moreover, the CRs achieved with all dose levels have been sustained over 6 months. Both FT596 and NKX019 demonstrated a favorable safety profile with no dose-limiting toxicity, GvHD, or neurotoxicity. These intriguing findings further strengthen the advantages of CAR-NK over CAR-T immunotherapy.

Regarding MM, there are four phase I, BCMA-targeted CAR-NK cell clinical trials registered, in which three of them initiated in China and one conducted in the US (**Table 3**). FT576, a BCMA-specific CAR-NK cellular product, has shown a glimpse of clinical activity in 3/9 patients enrolled. It is suggested that multiple doses of FT576 with/without daratumumab may offer a deeper and more sustained anti-MM response. These clinical results are indicative of the need of significant improvement in CAR-NK compared to CAR-T therapy to treat R/R MM patients.

Multiple clinical studies have reported disappointing results for adoptive autologous NK cell therapy in cancer treatment (150). However, when combined with hepatic arterial infusion chemotherapy, the autologous expanded and activated NK cells have shown exciting clinical efficacy (151). In a phase I study involving 11 evaluated patients, an objective response was observed in 7 patients (63.6%), with 4 (36.4%) achieving CR, and 3 (27.3%) had a partial response. Two patients had stable disease, while two had progressive disease, possibly due to NK cell dysfunction or exhaustion. The multi-dose product, Vax-NK/HCC, has earned therapeutic approval from the Korean Ministry of Food and Drug Safety for patients with locally advanced hepatocellular carcinoma who have exhausted other treatment options.

We consider TAK-007, a CAR-NK product co-developed by MD Anderson Cancer Center and Takeda, to be a leading contender, along with FT596 and NKX019, as the most promising CAR-NK cellular therapies for hematological malignancies. BCMA-targeted CAR NK cells, FT576, hold promise as one of the best NK cell therapies to eradicate MM.

## 5 Perspectives

Drawing parallels for CAR-NK from CAR-T cell therapy, the GMP-compliant manufacturing of CAR-NK also requires state-of-the-art cell processing centers and experienced medical workers. It should be noted that optimization of CAR such as titration and timing of CAR-mediated response, and metabolic engineering of NK cells is pivotal to overcome drug-resistant MM. Although the inherent anti-tumor functionality of NK cells makes them an optimal subject for CAR technology, prolonging the short lifespan and activation of the innate lymphocytes may help to generate a deep and durable anti-MM response. Since CAR-T cell therapy faces some challenges such as shedding of target antigens from surface and off-target effects in treating MM, CAR-NK cell approach may prevent the phenomenon by targeting multiple antigens, which are not capable of shedding under selection pressure, as well as incorporating homing factors into CAR. Off-the-shelf NK cell-based therapy is picking up steam to lower the cost of adoptive immune cell therapy and promote accessibility to broader range of patients with cancer. Differ from adoptive T cell therapy, multiple-dose administration of adoptive NK cells is necessary to induce deeper and more sustained anti-MM clinical response. Taken together, adoptive NK cells are an alternative and effective immune cell-based therapy that may improve clinical outcomes for MM patients.

## Author contributions

SV: Conceptualization, Visualization, Writing – original draft, Writing – review & editing. HP: Visualization, Writing – original draft. TP: Visualization, Writing – original draft. TL: Visualization, Writing – original draft. M-CV: Writing – original draft, Writing – review & editing. S-HJ: Writing – original draft, Writing – review & editing. J-JL: Writing – original draft, Writing – review & editing. X-HN: Conceptualization, Funding acquisition, Project administration, Supervision, Writing – original draft, Writing – review & editing.
